# Phytochemical Analysis and Biological Activity of *Astragalus onobrychis* : Quantitative Analysis of Phenolic Compounds, Antioxidants, and Antibacterial Activities

**DOI:** 10.1002/fsn3.70025

**Published:** 2025-01-31

**Authors:** Ibrahim Hosaflioglu

**Affiliations:** ^1^ Research Laboratory Practice and Research Center Igdir University Igdir Turkiye

**Keywords:** antioxidant, *Astragalus onobrychis*, phenolic compounds, quantitative analysis

## Abstract

Plants play an essential role in the food and pharmaceutical industries. Plants show broad‐spectrum biological activity due to the bioactive compound contents. Hence, plants provide an essential contribution to drug invention and progress. In this study, phytochemical analysis and antioxidant effects of the *Astragalus onobrychis* plant were investigated. The flower, leaf, and stem parts of *Astragalus onobrychis* were extracted with methanol. The quantitative analysis of corresponding parts was conducted by LC‐ESI‐MS/MS, and it was detected that the flowers, leaves, and stems contained 12, 19, and 17 compounds, respectively. The flowers were determined to contain kaempferol‐3‐glucoside (0.395 mg/g extract) as a major product. Additionally, routine (0.132) was defined as the leading product in the leaf parts of the plant. The main product of the stem part of the plant was detected as coumarin (0.068). Antioxidant activity tests of flower, leaf, and stem extracts of the *Astragalus onobrychis* plant were performed. It was determined that the flowers showed the highest antioxidants among them. DPPH activity of flowers was determined as 4.56 ± 0.42 (IC50, μg/mL). Moreover, the antibacterial activity of flowers, leaves, and stem was performed using *B*.*subtilis*, *S*. *aureus* , *E*. *coli* , and *P*. *aeruginosa* , and flowers displayed excellent antibacterial activity against *B*. *subtilis* and *E*. *coli* with a value of 10.5 μg/mL.

## Graphical abstract

The quantitative analysis of phenolic compounds in flowers, leaves, and stems was determined by LC‐ESI‐MS/MS. The flowers, leaves, and stems contain kaempferol‐3‐glucoside, routine, and coumarin as major products, respectively. The flowers showed the highest antioxidants. Moreover, the antibacterial activity of flowers, leaves, and stems was performed using *B*.*subtilis*, *S*. *aureus* , *E*. *coli* , and *P*. *aeruginosa* , and flowers displayed excellent antibacterial activity.

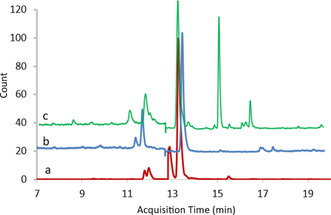

## 1 Introduction

Plants have been used for medicine and food since ancient times (Elmastas et al. 2004; Hadjra et al. 2023; Topçu et al. 1999). The development of spectroscopy and chromatography has made plants the subject of science (Demirtas et al. 2013). Metabolites found in plants form the basis of drug development (Sahin Yaglioglu et al. 2013). Additionally, secondary metabolites have inspired synthetic chemists to synthesize them (Lu et al. 2014). Intensive studies have been continued to isolate natural compounds from plants and to synthesize them in laboratories (Okten et al. 2015). In addition, natural compounds have been functionalized to yield semi‐synthetic compounds. Hence, it has been aimed at increasing biological and pharmaceutical efficiency (Newman and Cragg 2007).

Quantifying phenolic compounds in plants is crucial. Phenolic compounds play various roles in plant physiology, including defense against pathogens and herbivores, UV protection, and pigmentation. Quantifying phenolics helps researchers understand how plants respond to stressors and environmental cues (Erenler et al. 2023). Moreover, many phenolic compounds have antioxidant properties and are linked with health benefits in humans, such as reducing the risk of chronic diseases like cancer and cardiovascular disorders. Quantifying phenolics helps in assessing the nutritional and medicinal value of plant‐based foods and herbal medicines (Atalar et al. 2023). Quantifying phenolic compounds is essential for ensuring the quality and authenticity of agricultural products and food items. For instance, it can help detect adulteration, assess the freshness of produce, and determine the shelf life of processed foods (Erenler et al. 2015).

*Astragalus* L. genus belonging to the Leguminosae family is extensively distributed throughout the world, grown mainly in Europe, Asia, and North America. This genus is represented by 850 species. Astragalus species were reported to include saponins as major bioactive constituents. This genus has been used as folk medicine to treat different illnesses such as diabetes, respiratory infections, and leukemia (Sahin Yaglioglu et al. 2022).

Plant secondary metabolites can be categorized into several main groups based on their chemical structures and biosynthetic pathways (Elmastaş et al. 2015). Phenolics include flavonoids, phenolic acids, tannins, lignans, and lignins. They often contribute to the color, flavor, and antioxidant properties of plants. Terpenoids are derived from isoprene units and include compounds such as monoterpenes, sesquiterpenes, diterpenes, triterpenes, and steroids. They are diverse and play a role in defense, signaling, and attracting pollinators (Reddy, Odhav, and Bhoola 2003).

Alkaloids are nitrogen‐containing compounds with different structures and functions. Examples are caffeine, nicotine, morphine, and quinine. Alkaloids often have pharmacological effects and can act as repellents against herbivores. Glycosides consist of a sugar molecule (glycone) attached to a non‐sugar moiety (aglycone). They are widespread in plants and can have various biological activities, including antimicrobial, antifungal, and cytotoxic properties (Li and Vederas 2009).

Free radicals are extremely reactive molecules, including unpaired electrons (Gecer and Erenler 2023). Because of this unpaired electron, free radicals are unstable and seek to stabilize themselves by either donating or accepting an electron from other molecules, leading to a chain reaction of oxidative damage. These molecules are naturally produced in the body during various metabolic processes, such as cellular respiration and immune response. Additionally, they can be generated by external sources like exposure to UV radiation, pollution, tobacco smoke, and certain chemicals (Aissous et al. 2023). Antioxidants, including some phenolic compounds, and enzymes help neutralize free radicals by donating electrons without becoming destabilized themselves (Guzel et al. 2017). Consuming a diet rich in antioxidants or using antioxidant supplements may help mitigate the harmful effects of free radicals and reduce the risk of associated health problems. Consuming a diet rich in antioxidant‐rich foods is important for maintaining optimal health and reducing the risk of chronic diseases (Elmastas et al. 2018). Due to the bacterial resistance problem worldwide, there is a need to investigate new beneficial agents with broad‐spectrum antibacterial activity (Karan et al. 2024a).

In this study, quantitative analysis of phenolic compounds was determined from flowers, leaves, and stems of *Astragalus onobrychis* , and their antioxidant activities were determined using the DPPH, ABTS, and hydroxyl radical scavenging effects.

## 2 Material and Methods

### 2.1 Plant Materials

*Astragalus onobrychis* was collected from Igdir University campus and identified by taxonomic expert Dr. Belkis Muca Yigit, a voucher specimen was deposited in the Igdir University herbarium (No: INWM00000118).

### 2.2 Extraction of Plant Material

*Astragalus onobrychis* was collected, washed to remove dust and impurities, and dried in the shade. The flowers, leaves, and stems were separated. Each part was extracted in methanol for 24 h at room temperature. After the solvent was removed, the crude extract of each part of the *A. onobrychis* was yielded. They were kept in the fridge at +4 for usage (Khodja et al. 2023).

### 2.3 Quantitative Analysis of Natural Compounds

LC–MS/MS (Agilent Technologies) with A Poroshell 120 SB‐C18 column was employed for quantitative analysis of natural compounds in flowers, leaves, and stems of the *Astragalus onobrychis*. Each sample (50 mg) was dissolved in methanol (1.0 mL). After the addition of hexane, the mixture was centrifuged for 15 min. An aliquot (100 μL) was taken and diluted by adding methanol (450 μL) and water (450 μL). subsequently, after filtration (0.22 μm filter), the solution was applied to the device. Formic acid (0.1%) and ammonium formate (5.0 mM) in water A, formic acid (0.1%), and ammonium formate (5.0 mM) in methanol B were used as the mobile phase. The program was fixed as 0–5 min 50%, 6–12 min 70%, 13–25 min 90%, 26–30 min 5% for mobile phase B. The flow gas rate was adjusted as 11 L/min (Erenler, Karan, and Hosaflioglu 2023).

### 2.4 Antioxidant Activity

Antioxidant activity of flowers, leaves, and stems was conducted using ABTS, DPPH, and Hydroxyl radical scavenging assays. In ABTS radical scavenging assay, the stock solution of extract was prepared in methanol (1.0 mg/mL). The reaction was carried out in the phosphate buffer solution (500 mL). ABTS^•+^. The reaction of ABTS with sodium persulfate in the dark for 6 h at room temperature yielded the ABTS^•+^ radical cation solution. Later, the treatment was executed with the different concentrations of each sample (5–50 μg/mL) with the ABTS^•+^ solution.

In the DPPH assay, DPPH^•^ solution in ethanol (0.26 mM) was treated with the sample at different concentrations (5–50 μg/mL, 3.0 mL). The reaction mixture was incubated at room temperature for 30 min, and then absorbance measurement (517 nm) was executed. The results were calculated as IC_50_.

In hydroxyl radical assay, the mixture of hydrogen peroxide (40 mM, 1.0 mL), phosphate buffer solution (pH 7.4, 0.04 M, 2.4 mL), and sample (100 μL) was carried out and incubated for 10 min. The absorbance was measured at 230 nm.

### 2.5 Bacterial Culture

The flowers, leaves, and stem parts of *Astragalus onobrychis* were subjected to antibacterial activity using the minimum inhibition concentration (MIC) method against gram‐positive bacteria (*Bacillus subtilis, Staphylococcus aureus*), and gram‐negative bacteria (*Escherichia coli, Pseudomonas aeruginosa*) in Luria broth medium. Luria broth medium (1%) was autoclaved and added to the bacteria‐exposed slides, and agar (20 mg/mL) was used for solidification. The extracts of flowers, leaves, and stems were dissolved in deionized water. The sodium hydroxide was used to adjust the pH to 7.0–7.2. After the slides were incubated at 37°C for 18 h, the colonies were quantified (Karan et al. 2024b).

### 2.6 Minimum Inhibition Concentration

The MIC of flowers, leaves, and stem parts of *Astragalus onobrychis* was determined via double serial dilution in a liquid nutrient medium. Each extract was diluted with deionized water at a concentration of 168 μg/mL. The serial dilution was carried out to 10.50 μg/mL. The experiment was executed in a sterile, 96‐well plate. the Luria broth medium (5.0 mL) was used, and bacterial concentrations were 10^5^–10^6^ CFU/mL (cfu: colony forming units). Luria broth medium was a positive control. Inoculated broth was the negative control. After the medium was incubated at 37°C for 24 h, dilution was started. The sample (100 μL) was plated on LB agar plates and incubated at 37°C for 30 h, and then colonies were quantified. The minimal bacterial concentration was calculated as the lowest concentration that prevented the observable growth of the bacteria.

### 2.7 Statistical Analysis

The statistical analysis was executed by GraphPad Prism. ANOVA test was applied, and multiple comparisons were conducted with the Tukey test (*p* > 0.05). The results were presented with mean ± standard deviation.

## 3 Results and Discussion

**FIGURE 1:**
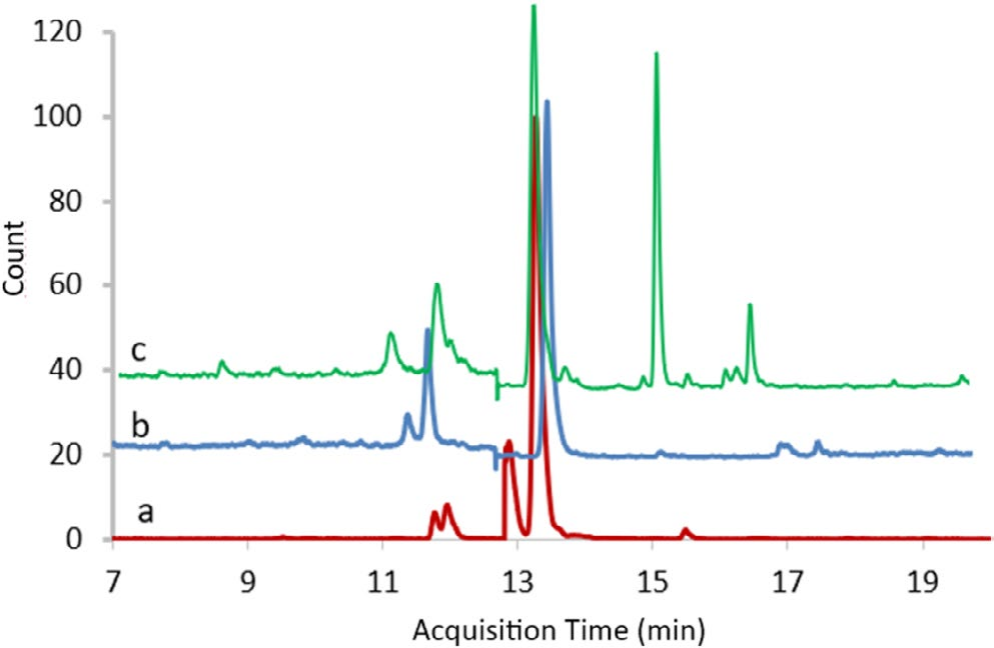
LC–MS/MS chromatogram of flowers (a), leaves (b) and stem (c).

**FIGURE 2:**
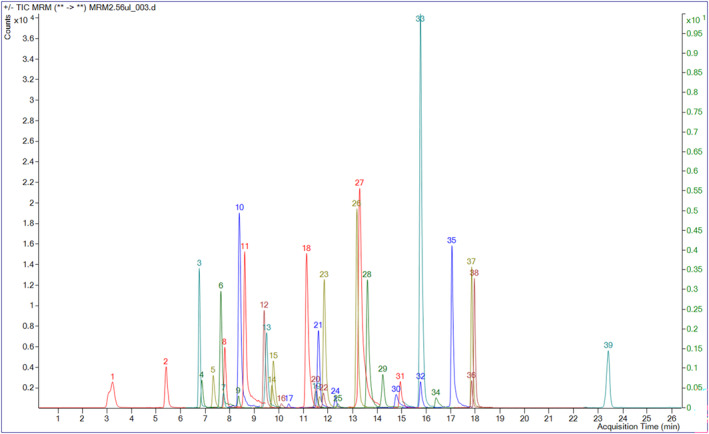
The MRM chromatogram of standard compounds by using LC–MS/MS (1‐Gallic acid, 2‐Protocatechuic acid, 3‐Epigallocatechin, 4‐Catechin, 5‐Chlorogenic acid, 6‐*4‐*Hydroxybenzaldehyde, 7‐Vanillic acid, 8‐Caffeic acid, 9‐Syringic acid, 10‐Caffeine, 11‐Vanillin, 12‐*p‐*Coumaric acid, 13‐Salicylic acid, 14‐Taxifolin, 15‐Resveratrol, 16‐*trans*‐ferulic acid, 17‐Sinapic acid, 18‐Scutellarin, 19‐*o‐*Coumaric acid, 20‐ Coumarin, 21‐ Protocatechuic ethyl ester, 22‐ Rutin, 23‐ Isoquercitrin, 24‐ Hesperidin, 25‐ Quercetin‐*3‐D‐*xyloside, 26‐ Kaempferol‐*3‐*glucoside, 27‐ Fisetin, 28‐ Baicalin, 29‐ *trans*‐Cinnamic acid, 30‐ Quercetin, 31‐ Naringenin, 32‐ Hesperetin 33‐ Morin, 34‐ Kaempferol, 35‐ Baicalein, 36‐ Luteolin, 37‐ Biochanin A, 38‐Chrysin, 39‐Diosgenin).

Quantitative analysis of phenolic compounds of flowers, leaves, and stems of *Astragalus onobrychis* plant was conducted using LC–MS/MS (Figure 1). The flowers were determined to contain kaempferol‐3‐glucoside (0.395 mg/g extract), isoquercitrin (0.081 mg/g extract), syringic acid (0.0086 mg/g extract) as major products. Moreover, rutin (0.132 mg/g extract), luteolin (0.121 mg/g extract), coumarin (0.062 mg/g extract), and hesperidin (0.047 mg/g extract) were determined as the main products in the leaf parts of the plant. The main products of the stem were detected as coumarin (0.068 mg/g extract), kaempferol‐3‐glucoside (0.049 mg/g extract), hesperetin (0.043 mg/g extract), syringic acid (0.041 mg/g extract) (Table 1). The standard compounds chromatograph is given in Figure 2.

**TABLE 1:** Quantitative analysis of natural compounds in the flowers, leaves, and stem of *Astragalus onobrychis* methanol extract (mg/g extract).

No	Compounds	RT	Flowers	Leaves	Stem
2	Shikimic acid	1.383	nd	nd	nd
3	Gallic acid	3.221	nd	0.003	0.003
4	Protocatechuic acid	5.467	nd	nd	nd
5	Epigallocatechin	6.815	nd	nd	nd
6	Catechin	6.904	nd	nd	0.001
7	Chlorogenic acid	7.443	nd	0.001	nd
8	Hydroxybenzaldeyde	7.697	nd	0.002	0.003
9	Vanillic acid	7.829	nd	nd	nd
10	Caffeic Acid	7.891	nd	nd	nd
11	Syringic acid	7.945	0.0086	0.022	0.041
12	Caffeine	8.498	0.0013	nd	nd
13	Vanillin	8.678	nd	0.002	0.005
14	o‐coumaric acid	9.495	0.0022	0.003	0.001
15	Salicylic Acid	9.871	0.0035	0.015	0.022
16	Taxifolin	9.670	nd	nd	nd
17	Resveratrol	9.874	nd	nd	nd
18	Polydatine	9.897	nd	nd	nd
19	Trans‐ferulic acid	10.182	nd	0.012	0.004
20	Sinapic acid	10.447	nd	0.003	0.004
21	Scutellarin	11.148	nd	nd	nd
22	p‐coumaric acid	11.607	nd	0.001	0.001
23	Coumarin	11.173	0.0056	0.062	0.068
24	Protocatehuic ethyl ester	11.676	nd	nd	nd
25	Hesperidin	11.906	0.0061	0.047	nd
26	Isoquercitrin	11.735	0.0814	0.011	0.027
27	Rutin	12.375	nd	0.132	nd
28	Quarcetin‐3‐xyloside	12.770	0.0043	nd	nd
29	Kaempferol‐3‐glucoside	13.282	0.395	0.006	0.049
30	Fisetin	13.293	0.0073	0.002	0.003
31	Baicalin	13.923	nd	nd	nd
32	Chrysin	14.255	nd	nd	nd
33	Daidzein	14.558	nd	nd	nd
34	Trans‐cinnamic acid	14.340	nd	nd	nd
35	Quercetin	14.931	nd	nd	nd
36	Naringenin	15.041	0.0012	0.003	0.004
37	Silibinin	15.800	nd	nd	nd
38	Hesperetin	15.848	0.0062	0.030	0.043
39	Morin	15.802	nd	nd	nd
40	Kaempferol	16.523	nd	0.010	0.013
41	Baicalein	17.101	nd	nd	nd
42	Luteolin	17.901	nd	nd	nd
43	Biochanin A	17.909	nd	nd	nd
44	Capcaicin	18.152	nd	nd	nd
45	Dihydrocapcaicin	18.716	nd	nd	nd
46	Diosgenin	23.533	nd	nd	nd

**FIGURE 3:**
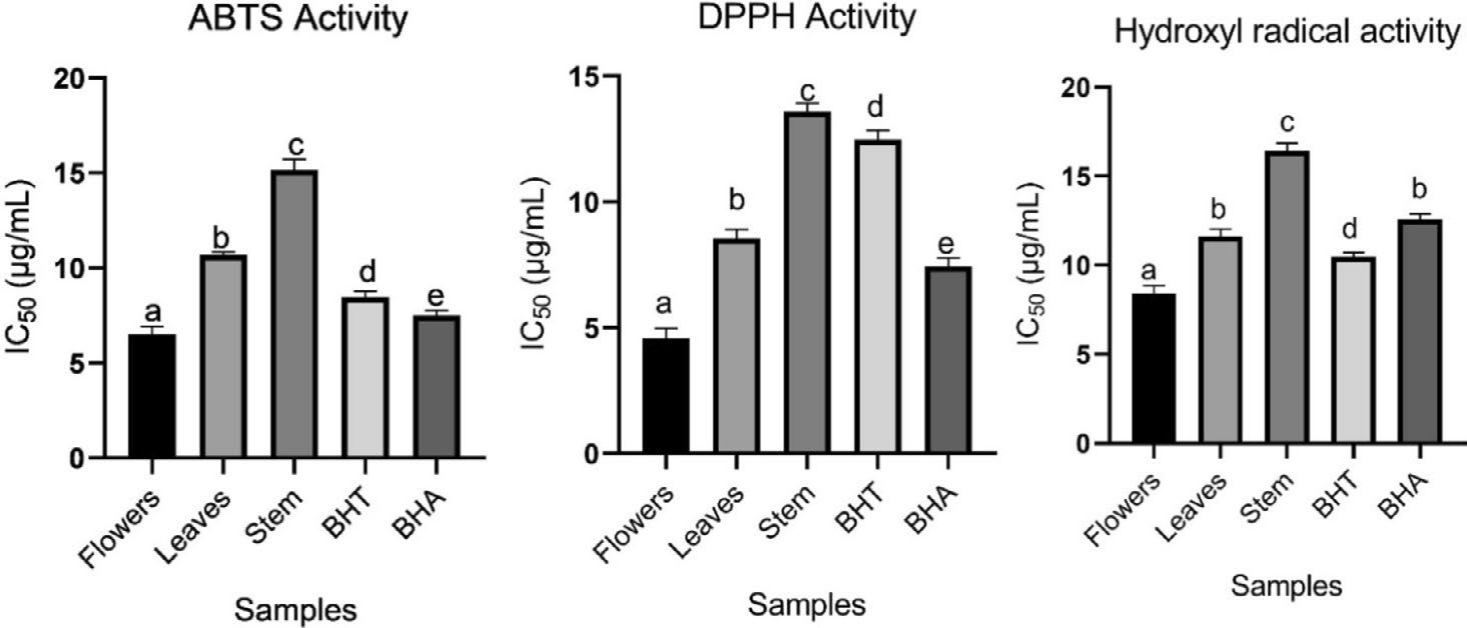
Antioxidant activity of *Astragalus onobrychis* (flowers, leaves, and stem). Different letters indicate a significant difference in means values. Values followed by the same letter are not significantly different (*p* < 0.05).

Antioxidant activity of *Astragalus onobrychis* (flowers, leaves, and stem) was conducted. In the ABTS^•+^ assay, flowers displayed outstanding activity (6.51 ± 0.42, IC_50_, μg/mL) in comparison to the leaves (10.69 ± 0.15, IC_50_, μg/mL), and stem (15.16 ± 0.56, IC_50_, μg/mL). The effect of standards, BHT, and BHA was observed as 8.47 ± 0.31 (IC_50_, μg/mL) and 7.50 ± 0.27 (IC_50_, μg/mL), respectively. In the DPPH assay, the same trend was observed. The flowers revealed higher activity with a value of 4.56 ± 0.42 (IC_50_, μg/mL) than that of the leaves (8.55 ± 0.36, IC_50_, μg/mL) and stem (13.59 ± 0.35, IC_50_, μg/mL). In the hydroxyl radical effect, the activity of flowers (8.44 ± 0.42, IC_50_, μg/mL) was detected to be higher than that of the leaves (11.60 ± 0.43, IC_50_, μg/mL) and stem (16.42 ± 0.43, IC_50_, μg/mL). The activity of BHT and BHA was determined as 10.49 ± 0.22 (IC_50_, μg/mL) and 12.57 ± 0.32 (IC50, μg/mL), respectively. The activity of flowers was measured higher than that of the leaves and stem (Figure 3). This is due to the bioactive compound content. Moreover, the synergic effect of compounds in the extract can lead to high activity. The plant's flowers include a high bioactive compound concentration that leads to antioxidant activity. In addition, kaempferol‐3‐glucoside, a major compound in flowers, was reported to show vigorous antioxidant activity (Calderon‐Montano et al. 2011).

Kaempferol is a natural compound abundant in medicinal plants. Kaempferol and its derivatives were reported to display considerable biological activity, including antioxidant, antimicrobial, anticancer, and antidiabetic properties (Jan et al. 2022).

Phenolics play essential roles in plant physiology, including defense against pathogens, UV protection, and pigmentation. Quantitative analysis helps in understanding the distribution and concentration of these compounds in different plant tissues and under varying environmental conditions. Many phenolic compounds possess bioactive properties with potential applications in medicine, food, and agriculture. Quantitative analysis helps identify and quantify these compounds, which is essential for their utilization in various fields. Many studies were carried out on medicinal plants that revealed considerable antioxidant activities. Moreover, plant‐based silver nanoparticles were reported to display excellent antioxidant effects (Erenler et al. 2021; Gecer 2021; Gecer et al. 2021; Rice‐Evans, Miller, and Paganga 1997).

Antibacterial effects of flowers, leaves, and stem of *Astragalus onobrychis* were carried out against the gram‐positive bacteria (*Bacillus subtilis*, *Staphylococcus aureus*), and gram‐negative bacteria (*Escherichia coli, Pseudomonas aeruginosa*) in Luria broth medium (Table 2). The MIC values of flower extract against *B. subtilis, S. aureus, E. coli, P. aeruginosa* were determined as 10.5, 21, 10.5, and 21 μg/mL, respectively. In addition, the MIC values of the leaf extract against *B. subtilis, S. aureus, E. coli, P. aeruginosa* were reported as 21, 42, 21, 42 μg/mL, respectively. However, the stem showed lower activity than that of the flowers and leaves. The MIC values of the stem against *B. subtilis, S. aureus, E. coli, P. aeruginosa* were determined as 42, 84, 84, 42 μg/mL, respectively. The standard amoxicillin MIC values were detected as 10.5, 21, 21, and 21 μg/mL for the flowers, leaves, and stems, respectively. The high concentration of bioactive compounds may lead to antibacterial activity.

**TABLE 2:** Antimicrobial activity MIC (μg/mL).

Isolates	Flowers	Leaves	Stem	Amoxicillin
*Bacillus subtilis*	10.5	21	42	10.5
*Staphylococcus aureus*	21	42	84	21
*Escherichia coli*	10.5	21	84	21
*Pseudomonas aeruginosa*	21	42	42	21

## 4 Conclusion

*Astragalus onobrychis* is an important plant consisting of valuable bioactive compounds. The flowers, leaves, and stems of this plant revealed good antioxidant activities as well as antibacterial effects. Among the flowers, leaves, and stems, the flowers consisted of the most bioactive compounds with the highest concentrations. Hence, the activity of flowers may be attributed to the high concentrations of bioactive compounds. This plant may be the main source of corresponding bioactive compounds.

## Author Contributions

**Ibrahim Hosaflioglu:** resources (lead), software (lead), supervision (lead), validation (lead), visualization (lead), writing – original draft (lead), writing – review and editing (lead).

## Conflicts of Interest

The author declares no conflicts of interest.

## Data Availability

The data that support the findings of this study are available on request from the corresponding author.
